# Gut Microbiota Dysbiosis and CIPN: State-of-the-Art Evidence and a Microbiota–Ozone Therapeutic Framework

**DOI:** 10.3390/cancers18132112

**Published:** 2026-06-29

**Authors:** Bernardino Clavo, Elizabeth Córdoba-Lanús, Gregorio Martínez-Sánchez, Ángeles Cánovas-Molina, Mario Federico, Saray Galván, Avinash Ramchandani-Vaswani, José E. Piñero, Carla Antonilli, Gretel Benítez, Luis Cobiella-Hernández, David Pérez-Rodríguez, Carmen Pérez-Santana, Ruth Martín-Alfaro, Maria Fernández-Tagarro, Juan A. Díaz-Garrido, Jesús M. González-Martín, Rocío Martínez-Pérez, Jacob Lorenzo-Morales, Francisco Rodríguez-Esparragón

**Affiliations:** 1 Research Unit, Hospital Universitario de Gran Canaria Dr. Negrín, 35019 Las Palmas de Gran Canaria, Spain; mcanmol@gobiernodecanarias.org (Á.C.-M.); mfederi@gobiernodecanarias.org (M.F.); lcobiella@fciisc.es (L.C.-H.); dperez@fciisc.es (D.P.-R.); carmen.perez118@alu.ulpgc.es (C.P.-S.); jdiagarb@gobiernodecanarias.org (J.A.D.-G.); jmgonmar@gobiernodecanarias.org (J.M.G.-M.); frodesp@gobiernodecanarias.org (F.R.-E.); 2 Chronic Pain Unit, Hospital Universitario de Gran Canaria Dr. Negrín, 35019 Las Palmas de Gran Canaria, Spain; 3 Radiation Oncology Department, Hospital Universitario de Gran Canaria Dr. Negrín, 35019 Las Palmas de Gran Canaria, Spain; 4 Instituto de Investigación Sanitaria de Canarias (IISC), 35019 Las Palmas de Gran Canaria, Spain; gregorcuba@yahoo.it; 5 University Institute for Research in Biomedicine and Health (UIBS), Molecular and Translational Pharmacology Group, University of Las Palmas de Gran Canaria, 35016 Las Palmas de Gran Canaria, Spain; 6 Instituto Universitario de Enfermedades Tropicales y Salud Pública de Canarias, Universidad de La Laguna, 38296 San Cristóbal de La Laguna, Spain; acordoba@ull.edu.es (E.C.-L.); jpinero@ull.edu.es (J.E.P.); 7 CIBER de Enfermedades Infecciosas (CIBERINFEC), Instituto de Salud Carlos III, 28029 Madrid, Spain; 8 Spanish Group of Clinical Research in Radiation Oncology (GICOR), 28290 Madrid, Spain; 9 Department of Biochemistry, Microbiology, Cellular Biology and Genetics, Universidad de La Laguna, 38200 San Cristóbal de La Laguna, Spain; 10 Independent Research, 60126 Ancona, Italy; 11 Medical Oncology Department, Hospital Universitario de Gran Canaria Dr. Negrín, 35019 Las Palmas de Gran Canaria, Spain; sgalrui@gobiernodecanarias.org (S.G.); pantoniyv@gobiernodecanarias.org (C.A.); 12 Medical Oncology Department, Complejo Hospitalario Universitario Insular Materno-Infantil de Gran Canaria, 35016 Las Palmas de Gran Canaria, Spain; aramvas@gobiernodecanarias.org (A.R.-V.); gbenlop@gobiernodecanarias.org (G.B.); 13 Department of Obstetrics and Gynecology, Pediatrics, Preventive Medicine and Public Health, Toxicology, Legal and Forensic Medicine, and Parasitology, Universidad de La Laguna, 38200 San Cristóbal de La Laguna, Spain; 14 Clinical Analysis Department, Hospital Universitario de Gran Canaria Dr. Negrín, 35019 Las Palmas de Gran Canaria, Spain; rmaralf@gobiernodecanarias.org (R.M.-A.); mfertag@gobiernodecanarias.org (M.F.-T.); 15 Department of Psychiatry, Hospital Universitario de Gran Canaria Dr. Negrín, 35019 Las Palmas de Gran Canaria, Spain; 16 Hospital Universitario de Gran Canaria Dr. Negrín, Universidad Fernando Pessoa Canarias, 35019 Las Palmas de Gran Canaria, Spain; 17 Arganda Heath Center La Felicidad, 2, 28500 Arganda del Rey, Spain; rocio.martinez.perez@salud.madrid.org

**Keywords:** gut microbiota, dysbiosis, chemotherapy-induced peripheral neuropathy, gut–nerve axis, ozone therapy, rectal ozone insufflation, microbiota modulation, cancer treatment toxicity, neuroinflammation, supportive oncology

## Abstract

Background/Objectives: Chemotherapy-induced peripheral neuropathy (CIPN) affects up to 85% of patients receiving neurotoxic regimens, often leading to dose reduction and impaired quality of life, yet effective preventive or therapeutic options remain scarce. Emerging evidence implicates chemotherapy-induced gut microbiota dysbiosis in CIPN pathogenesis via a gut–nerve axis. Concurrently, rectal ozone insufflation (ROI) has been shown to modulate the gut microbiota and reduce inflammation in preclinical models. This article critically examines the evidence on the role of gut dysbiosis in CIPN, evaluates the microbiota-modulating capacity of rectal ozone therapy (OT), and assesses the biological plausibility of ozone as a microbiota-targeting intervention for CIPN, while explicitly distinguishing between established evidence and hypothetical mechanisms. Evidence synthesis: Neurotoxic agents induce dysbiosis marked by reduced microbial diversity, loss of short-chain fatty acid-producing bacteria, and expansion of pro-inflammatory taxa. Preclinical models demonstrate a causal role for specific microbial communities in CIPN, with microbiota depletion or fecal transplantation modulating neuropathic phenotypes. In human cohorts, dysbiosis severity correlates with CIPN symptoms. Preclinical studies show that ROI restores microbial balance, enhances short-chain fatty acid levels, and strengthens intestinal barrier function via Nrf2/HO-1 and SIRT1 pathways. Preliminary retrospective data from small case series (*n* = 7 and *n* = 15) report sustained symptom improvement in CIPN patients receiving OT. However, no human study has directly linked ozone-induced microbiota changes to clinical outcomes, and the clinical evidence for OT in CIPN remains limited to uncontrolled observations. Conclusions: Convergent preclinical evidence supports a biological rationale for investigating ROI as a microbiota-targeting intervention in CIPN. However, this rationale remains largely hypothetical in the clinical setting. High-quality randomized controlled trials with longitudinal microbiome profiling are urgently needed to establish mechanistic causality and to determine whether the promising preclinical findings translate into clinically meaningful benefits. Until such evidence is available, the framework presented here should be regarded as hypothesis-generating rather than as a basis for clinical practice.

## Simple Summary

Chemotherapy often causes a painful and altered nerve condition called chemotherapy-induced peripheral neuropathy, forcing many cancer patients to reduce or stop their treatment. Unfortunately, few therapies are available to prevent or treat this condition. Recent research shows that damage to the bacteria living in our gut—caused by chemotherapy—can trigger nerve inflammation and worsen neuropathic pain. This review explores a working hypothesis: that delivering medical ozone through rectal insufflation could potentially help by restoring healthy gut bacteria, repairing the intestinal barrier, and reducing inflammation. However, it is important to emphasize that the clinical evidence supporting this approach is currently limited to small, uncontrolled case series, and the proposed mechanisms remain largely hypothetical. The authors aim to summarize the existing evidence linking gut bacteria to chemotherapy-induced neuropathy and to explore whether ozone therapy, as a microbiota-modulating strategy, warrants investigation as a potential new approach to managing this debilitating side effect. The findings are intended to generate hypotheses and guide the design of future clinical trials, rather than to provide clinical recommendations.

## 1. Introduction

Chemotherapy-induced peripheral neuropathy (CIPN) is a frequent, often persistent toxicity of many frontline cytotoxic regimens, with major implications for dose intensity, survival, and quality of life [1]. Growing evidence links CIPN to chemotherapy-driven gut microbiota dysbiosis and gut–nerve crosstalk [2], highlighting the microbiota–brain axis as a new therapeutic target, to which redox- and immunity-modulating strategies such as ozone therapy (OT) may be conceptually relevant [3,4].

CIPN is a predominantly sensory peripheral neuropathy with numbness, tingling, burning pain, and “glove and stocking” distribution; motor and autonomic involvement occur in some patients [5]. Symptoms may emerge during treatment and persist in ~30% of patients for ≥1 year after chemotherapy, substantially impairing function and quality of life [5,6]. Mechanistically, CIPN reflects axonal degeneration, microtubule disruption, mitochondrial dysfunction, altered ion channel expression, and neuroinflammation in peripheral and central pathways [1].

Across neurotoxic agents (platinums, taxanes, vinca alkaloids, bortezomib, thalidomide), an estimated 19–85% of patients develop CIPN during treatment [1]. CIPN is a major dose-limiting toxicity, often necessitating dose reduction, delay, or premature discontinuation, with potential compromise of treatment efficacy and survival [7]. Long-term CIPN contributes substantially to survivorship burden and healthcare costs [8].

Guidelines consistently conclude there is no effective pharmacologic prevention of CIPN; several proposed agents are discouraged [5,7]. For established painful CIPN, duloxetine is the only drug with moderate evidence and guideline endorsement; however, its analgesic benefit is modest, appears to be maintained only during treatment, may be associated with adverse effects, and many patients remain symptomatic [9,10]. Other pharmacologic and non-pharmacologic approaches (gabapentinoids, tricyclics, topical agents, neuromodulatory and rehabilitative strategies) show inconsistent or limited efficacy and lack robust evidence [9]. This therapeutic gap supports exploration of novel disease-modifying strategies.

Multiple reviews and preclinical studies now implicate the microbiota–gut–brain axis in CIPN pathogenesis. Chemotherapy disrupts gut microbial composition and barrier integrity, promoting dysbiosis, systemic leakage of microbial products, and immune activation [11,12]. Dysbiotic microbiota can drive glial and immune cell activation, neuroinflammation, and spinal microgliosis, contributing to neuropathic pain [11,12,13,14]. Antibiotic-induced microbiota depletion or fecal microbiota transplantation (FMT) modulate chemotherapy-induced mechanical and thermal hypersensitivity and associated cytokine profiles in rodent models, underscoring a causal role for gut microbes in CIPN [13,15].

Chemotherapeutic agents such as oxaliplatin, paclitaxel, and cisplatin induce characteristic shifts in gut microbiota, including altered *Bacteroidetes*/*Firmicutes* ratios and depletion of barrier-supporting taxa such as *Akkermansia muciniphila* [15]. These changes are associated with increased intestinal permeability, enhanced translocation of lipopolysaccharide (LPS), and activation of Toll-like receptor 4 (TLR4)-dependent inflammatory cascades in dorsal root ganglia (DRG) and spinal cord [13,15]. Blocking or attenuating dysbiosis—via antibiotics, probiotics, FMT, or other microbiota-targeted manipulations—reduces macrophage and microglial activation, pro-inflammatory cytokines (e.g., IL-6, TNF-α), and behavioral signs of neuropathic pain in animal models [15]. Collectively, these data position chemotherapy-induced dysbiosis as a central upstream driver of neuroimmune mechanisms in CIPN.

Given the established role of gut microbiota dysbiosis in the pathogenesis of CIPN—driving neuroinflammation, immune–glial activation, and barrier dysfunction—therapeutic strategies aimed at restoring microbial homeostasis represent a promising but underexplored avenue [15]. OT, particularly through rectal insufflation (ROI), offers a compelling biological rationale in this context [16]. Beyond its well-recognized pleiotropic effects on redox signaling, tissue oxygenation, and immune modulation, OT has been shown to modify the intestinal mucosal oxidative balance, microcirculation, and local immune milieu [17]. Recent preclinical evidence suggests that ROI can positively influence gut microbial ecosystems [4,18]. Consequently, OT emerges as a potential microbiota-modulating intervention that may simultaneously address two interconnected drivers of CIPN: dysbiosis and neuroinflammation. This mechanistic convergence—coupled with the current lack of effective preventive strategies for CIPN—strongly justifies exploring OT as an adjunctive, biology-targeted tool to mitigate chemotherapy-induced neurotoxicity through optimization of the gut–nerve axis [19].

This narrative review aims to provide a comprehensive and updated overview of the pathophysiological mechanisms underlying CIPN, current therapeutic limitations, and emerging strategies targeting key biological pathways. Particular emphasis is placed on the role of gut microbiota dysbiosis as a mechanistic contributor to neurotoxicity and as a potential therapeutic target, as well as on the rationale for microbiota-modulating interventions.

In this context, the present work builds upon our previous review on OT in CIPN [20], extending its scope to specifically address microbiota-related mechanisms. It also complements our ongoing clinical research protocol [19], which investigates the modulation of gut microbiota through ROI in patients with radiotherapy/chemotherapy-induced pelvic toxicity. By integrating these lines of research, this review proposes a translational framework linking microbiota alterations, systemic and neuroinflammatory pathways, and the potential role of OT as a microbiota-modulating intervention in CIPN.

Before proceeding, it is important to state explicitly that the integrative model proposed here—linking ozone therapy, gut microbiota modulation, and CIPN improvement through the gut–nerve axis—remains a working hypothesis. While each mechanistic component is supported by independent lines of evidence from preclinical and clinical studies, the complete sequence from ozone administration through microbiota restoration to measurable neurological benefit has not yet been tested as an integrated pathway in any single study. The value of this article lies not in asserting proven efficacy, but in providing a coherent, hypothesis-generating framework to guide future experimental validation and to identify the most critical knowledge gaps that must be addressed before any clinical recommendation can be considered.

## 2. Materials and Methods

### 2.1. Search Strategy and Information Sources

A comprehensive literature search was conducted to identify relevant studies examining the relationship between gut microbiota dysbiosis, CIPN, and OT. The following electronic databases were searched from inception through March 2026: PubMed/MEDLINE, Scopus, Web of Science, and Google Scholar for grey literature. The search strategy combined controlled vocabulary (MeSH terms where applicable) and free-text keywords, including: “chemotherapy-induced peripheral neuropathy” OR “CIPN” OR “neuropathic pain” AND “gut microbiota” OR “dysbiosis” OR “microbiome” AND “ozone therapy” OR “rectal ozone insufflation” OR “medical ozone” AND “gut–nerve axis” OR “microbiota–gut–brain axis”. Additional studies were identified by manual screening of reference lists of included articles and relevant systematic reviews.

### 2.2. Eligibility Criteria

Preclinical studies (in vivo animal models), clinical reports (including retrospective studies, case series, and prospective observational studies), systematic reviews, and narrative reviews published in peer-reviewed journals were considered eligible if they addressed at least one of the following: (i) the impact of neurotoxic chemotherapeutic agents (taxanes, platinum compounds, vinca alkaloids) on gut microbiota composition; (ii) the mechanistic role of gut microbiota dysbiosis in CIPN pathogenesis via the gut–nerve axis; (iii) the effects of ROI on gut microbiota modulation, intestinal barrier function, or inflammatory pathways; or (iv) clinical outcomes of OT in patients with CIPN or related neuropathic conditions. No restrictions on language or publication date were applied. Conference abstracts and unpublished data were excluded.

### 2.3. Data Synthesis and Presentation

Given the heterogeneity of study designs, outcome measures, and experimental models, a narrative synthesis approach was adopted. Evidence was organized thematically according to the three predefined objectives: (1) the pathogenic role of gut microbiota dysbiosis in CIPN; (2) the microbiota-modulating capacity of ROI; and (3) the biological plausibility of OT as a microbiota-targeting intervention for CIPN. Key findings were extracted and summarized descriptively, with particular attention to causal evidence from fecal microbiota transplantation studies, preclinical mechanistic data, and preliminary clinical outcomes.

### 2.4. Data Availability Statement

All data presented in this review are derived from previously published studies cited in the reference list. No new primary data, custom computer code, or proprietary materials were generated or analyzed during this study. The search strategy and inclusion criteria are fully disclosed to enable replication. The corresponding author may be contacted for additional documentation or clarification regarding the synthesis process.

### 2.5. Ethical Approval

As this manuscript is a narrative review of published literature and does not involve direct human or animal subjects, primary ethical approval was not required. All included studies cited in this review had obtained appropriate institutional ethics approval as reported in their respective original publications.

### 2.6. Generative Artificial Intelligence Disclosure

During the preparation of this work, the authors used generative artificial intelligence (GenAI) solely for superficial text editing purposes, including grammar correction, spelling checks, punctuation standardization, and formatting consistency. No GenAI tools were used to generate substantive content, data, tables, figures, or interpretative analysis, nor were they employed in study design, literature search, data extraction, or conclusion formulation. The authors assume full responsibility for the accuracy, originality, and integrity of all content presented in this manuscript.

## 3. Pathophysiology of CIPN

CIPN results from the convergence of direct neurotoxicity, neuroinflammation, and oxidative stress/mitochondrial damage. These mechanisms, common to taxanes, platinum compounds, and vinca alkaloids (Table 1), produce a distal “glove-and-stocking” sensory neuropathy characterized by axonal degeneration and neuropathic pain [1,21,22].

Neurotoxic mechanisms of chemotherapy involve:

Distal axonal damage and axonal transport: Disruption of microtubules interferes with anterograde and retrograde axonal transport, leading to distal degeneration of axonal segments and membrane remodeling [21,23].

Mitochondrial dysfunction and oxidative stress: (a) Taxanes, platinum compounds, vinca alkaloids, and bortezomib induce axonal mitotoxicity, mitochondrial swelling and vacuolization, opening of the permeability transition pore, cytochrome c release, and neuronal apoptosis [1,22]. (b) Mitochondrial damage increases reactive oxygen species (ROS) production, resulting in bioenergetic failure and neuropathy progression [24].

### 3.1. Neuroinflammation in CIPN

CIPN is associated with immune processes and neuroinflammation in the peripheral nerves, DRG, and spinal cord [22,23]. Chemotherapy activates microglia and astrocytes in the dorsal spinal cord, as well as macrophages/lymphocytes in the nerves and DRG, with subsequent cytokine production [23]. Levels of TNF-α, IL-1β, IL-6, and other chemokines increase, while anti-inflammatory cytokines (IL-10, IL-4) decrease, creating a pronociceptive environment [25]. These mediators enhance the excitability of DRG and dorsal horn neurons (peripheral and central sensitization), contributing to allodynia and hyperalgesia [7].

### 3.2. Oxidative Stress and Redox Imbalance

Oxidative stress is a central mechanism: damaged mitochondria generate excessive ROS, causing damage to neuronal lipids, proteins, and DNA [22]. The peripheral nervous system is particularly vulnerable due to its high phospholipid content and weak antioxidant defenses [24]. Dysfunction of antioxidant systems (including dysregulation of nuclear factor erythroid 2-related factor 2 (Nrf2) and enzymes such as superoxide dismutase (SOD) and glutathione-S-transferases) is linked to neurotoxicity from cisplatin and other agents [26].

CIPN arises from the interplay of distal axonal damage mediated by microtubule disruption, mitochondrial dysfunction with oxidative stress, and neuroinflammation driven by cytokines and glial/immune cells. These processes shared, albeit with nuances, among taxanes, platinum compounds, and vinca alkaloids, converge to produce a long-lasting, painful, and often persistent sensory neuropathy.

## 4. Gut Microbiota and Chemotherapy-Induced Dysbiosis

### 4.1. Baseline Role of Gut Microbiota in Host Homeostasis

In the eubiotic state, the gut microbiota maintains a diverse and symbiotic community that supports multiple physiological functions [27,28]:

- Short-chain fatty acids (SCFAs). Microbial fermentation of dietary fiber produces acetate, propionate, and butyrate. These metabolites regulate intestinal barrier integrity by upregulating tight junction proteins, including occluding claudin-1, and zonula occludens (ZO-1). Butyrate serves as the primary energy source for colonocytes and exerts anti-inflammatory effects through inhibition of histone deacetylases (HDACs) and suppression of Nuclear Factor kappa B (NF-κB) and NLRP3 inflammasome signaling [27,29].
- Intestinal barrier integrity. SCFAs, along with indole derivatives and secondary bile acids, maintain epithelial barrier function. Acetate produced by *Bifidobacterium* spp. enhances barrier protection and epithelial defense. Disruption of this barrier is associated with increased systemic inflammation and susceptibility to infection [27,29].
- Immune modulation. Gut microorganisms interact with mucosal immune cells at the gut-associated lymphoid tissue (GALT) and mesenteric lymph nodes, providing signals (pathogen-associated molecular patterns (PAMPs), damage-associated molecular patterns (DAMPs)) to antigen-presenting cells that modulate both local and systemic immunity. SCFAs signal through G protein-coupled receptors (GPCRs) such as GPR43, GPR41, and GPR109A to control immune homeostasis, promote regulatory T cell differentiation, and reduce inflammatory cytokine expression [27,30].

### 4.2. Chemotherapy-Induced Dysbiosis

Chemotherapy produces profound compositional and functional alterations in the gut microbiota [31] as shown in Table 2.

- Decreased alpha diversity. In patients receiving oral fluoropyrimidines, alpha diversity decreased significantly, particularly in those who developed diarrhea. Studies in myeloablative chemotherapy (without concomitant antibiotics) confirmed severe reductions in diversity [35].
- Loss of SCFA-producing bacteria. Chemotherapy consistently reduces *Firmicutes* (including *Faecalibacterium* and *Roseburia*) and *Actinobacteria* (including *Bifidobacterium*). In a study of 28 patients with non-Hodgkin’s lymphoma, *Firmicutes* and *Actinobacteria* decreased significantly (*p* = 0.0002) following chemotherapy. In patients with chemotherapy-induced diarrhea, *Bifidobacterium* abundance decreased significantly (*p* = 0.019), while it increased in those without diarrhea [35,36,37].
- Expansion of pathobionts. *Proteobacteria* increased significantly (*p* = 0.0002) after chemotherapy, particularly Enterobacteriaceae. Chemotherapy-induced epithelial cell death releases purine-containing metabolites that drive transcriptional reprogramming of Enterobacteriaceae, promoting their respiration and purine utilization-dependent expansion [36,37].
- Metabolic alterations. Dysbiosis is associated with reduced capacity for nucleotide metabolism (*p* = 0.0001), energy metabolism (*p* = 0.001), and metabolism of cofactors and vitamins (*p* = 0.006), with increased glycan metabolism and xenobiotic biodegradation. Metabolites such as TMAO (trimethylamine N-oxide), produced by certain bacteria, have been associated with tumor promotion in various cancer types [38,39].

### 4.3. Intestinal Barrier Dysfunction

- “Leaky gut” (increased intestinal permeability). Chemotherapeutic agents directly and rapidly damage proliferating intestinal epithelial cells, compromising tight junctions and increasing paracellular permeability. In murine models, among common agents, oxaliplatin causes the most severe intestinal damage in murine models, followed by 5-FU and irinotecan. Loss of SCFA production exacerbates barrier dysfunction: oral butyrate improved epithelial permeability and prevented irinotecan-induced increases in β-glucuronidase activity [40,41].
- Bacterial translocation. Barrier disruption permits passage of bacteria and their products from the intestinal lumen into mesenteric lymph nodes and secondary lymphoid organs. Cyclophosphamide treatment was associated with translocation of Gram-positive bacteria such as *Enterococcus hirae* and *Lactobacillus johnsonii* to the spleen and lymph nodes, stimulating Th17 and Th1 immune responses. This phenomenon has a dual role: it may contribute to antitumor efficacy but also to systemic toxicity [41].
- Systemic immune activation. Bacterial translocation and release of LPS and other pathogen-associated molecular patterns (PAMPs) activate inflammatory pathways including TLR4/MyD88/NF-κB and NOD/RIP2/NF-κB, with production of proinflammatory cytokines (TNF-α, IL-1β, IL-6, IFN-γ). Chemotherapy-associated dysbiosis correlates with gastrointestinal toxicity, neutropenia, and even cardiotoxicity. In the context of CAR-T cell therapy, prior dysbiosis (from broad-spectrum antibiotics) was associated with higher incidence of cytokine release syndrome [42].

Collectively, chemotherapy generates a vicious cycle: direct epithelial damage promotes dysbiosis, dysbiosis reduces production of protective SCFAs, which aggravates barrier dysfunction and facilitates bacterial translocation with systemic immune activation, perpetuating inflammation and gastrointestinal toxicity [43].

## 5. The Gut–Nerve Axis in CIPN

### 5.1. Mechanistic Pathways: Microbiota–Nervous System Signaling

The gut microbiota communicates with the peripheral and central nervous systems through three principal routes that are relevant to CIPN:

Immune pathways: Chemotherapy-induced dysbiosis increases intestinal permeability, allowing bacterial products, particularly LPS, to enter systemic circulation. LPS activates TLR4 on DRG neurons, satellite glial cells, and spinal cord glia, triggering downstream MyD88/NF-κB and MAPK (ERK1/2, p38) signaling cascades that produce proinflammatory cytokines (TNF-α, IL-1β, IL-6) and chemokines (MCP-1/CCL2) [12,44]. This immune-mediated signaling recruits macrophages into the DRG, which further amplifies neuroinflammation and contributes to intraepidermal nerve fiber loss [45]. Additionally, the DAMP molecule high mobility group box-1 (HMGB1) is released during chemotherapy-induced tissue injury and activates both TLR4 and RAGE receptors on spinal microglia, upregulating proinflammatory cytokines during the early stages of neuropathic pain [46]. In long-term germ cell tumor survivors, elevated plasma HMGB1 (a marker of gut microbial translocation) was associated with worse CIPN symptom burden [47].

Metabolic pathways: Gut microbiota-derived metabolites directly modulate nociceptive signaling. Chemotherapy-induced expansion of Clostridium species increases production of deoxycholic acid (DCA), a secondary bile acid. DCA elevates serum CCL5 and induces CCR5 overexpression in DRG neurons through the bile acid receptor TGR5, contributing to neuronal hyperexcitability. Notably, the CCR5 antagonist maraviroc suppressed paclitaxel-induced neuropathic pain in this model [48]. Conversely, loss of SCFA-producing bacteria reduces circulating butyrate and propionate, removing their anti-inflammatory and neuromodulatory protective effects [34].

Vagal pathway (emerging evidence, with caveats). The vagus nerve represents a direct neural route for gut-to-brain communication. *Roseburia* intestinalis-derived butyrate has been shown to activate vagal neurons through GPR41 receptors, enhancing vagal neurotransmission to the nucleus tractus solitarius (NTS) and suppressing the central amygdala (CeA), a brain region involved in pain perception. Vagal knockout of Gpr41 abolished these analgesic effects [49]. However, this evidence derives from postherpetic neuralgia models rather than CIPN specifically, and the relevance of vagal signaling to CIPN, which primarily involves peripheral sensory neurons and DRG, remains to be established. The gut microbiota’s intersection between the microbiome–gut–brain axis and the neuroimmune–endocrine axis forms a complex network that can directly or indirectly affect key components involved in CIPN manifestations [50].

### 5.2. Role of SCFAs

Butyrate as an anti-inflammatory and epigenetic modulator. Sodium butyrate (NaB) has demonstrated significant neuroprotective effects in CIPN models through multiple mechanisms:

- Anti-inflammatory effects. In an oxaliplatin-induced peripheral neuropathy (OIPN) rat model, NaB significantly decreased mechanical and cold allodynia scores and reduced sciatic nerve TNF-α levels while increasing IL-10 and nerve growth factor (NGF) expression. NaB also increased immunohistochemical expression of FOXP-3 (regulatory T cell marker) and PPAR-γ, indicating immunomodulatory and anti-inflammatory properties. Prophylactic NaB (before OIPN induction) showed greater neuroprotection than concurrent administration [51].
- Epigenetic regulation via HDAC inhibition. Butyrate acts as a potent HDAC inhibitor. In bone cancer pain models, butyrate supplementation decreased HDAC2 expression in the spinal dorsal horn and increased μ-opioid receptor (MOR) expression, potentiating morphine analgesia. In chronic constriction injury models, sodium butyrate (200–400 mg/kg oral, 14 days) attenuated cold allodynia, mechanical allodynia, and thermal hyperalgesia, with concurrent reduction in sciatic nerve TNF-α. Butyrate also increased expression of PPAR-α and PPAR-γ receptors while reducing COX-2, iNOS, TNF-α, and neuronal activation marker c-Fos, with analgesic effects mediated partly through ATP-dependent K^+^ channels [52].

Impact on neuropathic pain in CIPN. Propionic acid has also been shown to suppress nociceptive neuronal excitability via GPR41 signaling-mediated inhibition of voltage-gated Ca^2+^ channels in the central terminals of spinal nociceptive neurons [53].

### 5.3. Microbiota-Driven Neuroinflammation

LPS/TLR4/neuroinflammation cascade. TLR4 has emerged as a central mediator linking gut-derived endotoxemia to CIPN. Key findings include:

- TLR4 expression increases in DRG neurons as early as day 1 after oxaliplatin treatment and persists through day 14. Co-treatment with TLR4 antagonists (LPS-RS) or minocycline attenuated hyperalgesia and blocked downstream MyD88 and TRIF signaling [54].
- TLR4 antisense oligodeoxynucleotides prevented CIPN development induced by all three major drug classes (oxaliplatin, paclitaxel, bortezomib) and reversed established CIPN from oxaliplatin and paclitaxel [44].

Glial activation. Both peripheral and central glial cells are critically involved: (a) Spinal microglia proliferate in paclitaxel-treated mice harboring pain-sensitive microbiota (C57BL/6) but not in those with pain-resistant microbiota (129SvEv), directly linking gut microbial composition to central glial activation [8]. (b) Astrocytes in the spinal dorsal horn show increased GFAP expression in CIPN. FMT from healthy donors reduced astrocytic GFAP expression and TLR4/p38MAPK pathway activation in both the colon and spinal cord of paclitaxel-treated rats [55]. (c) Paclitaxel activates TLR4 on spinal astrocytes (but not microglia or neurons in the spinal cord), sensitizing TRPV1 receptors and impairing capsaicin-induced tachyphylaxis, leading to sustained nociceptive hyperactivity. Sustained minocycline preincubation mitigated this sensitization [56]. (d) In the DRG, TLR4 activation leads to MCP-1 upregulation, macrophage infiltration, TNF-α production, and ultimately loss of intraepidermal nerve fibers, a hallmark of clinical CIPN [45].

Peripheral nerve fiber-mediated mechanisms. While the DRG represents a primary site of microbiota–nerve interaction due to its lack of a complete blood–nerve barrier (BNB), emerging evidence indicates that gut-derived mediators also act directly on peripheral nerve structures. Schwann cells express functional TLR4 and produce pro-inflammatory cytokines (TNF-α, IL-1β) upon LPS stimulation in a TLR4-dependent manner, functioning as sentinel immune cells in the PNS analogous to microglia in the CNS [57]. Dysbiosis-induced systemic LPS elevation has been proposed to drive Schwann cell-mediated TNF-α production as a key mechanism in neuropathy development [58]. Importantly, the mechanistic chain extends to distal nerve terminals: TLR4-driven macrophage infiltration into the DRG leads to loss of intraepidermal nerve fibers (IENFs), the pathological hallmark of clinical CIPN, and inhibition of this cascade (via clodronate or TLR4 antagonism) prevents IENF loss. The relative permeability of the BNB, particularly at the DRG and distal nerve endings, renders peripheral nerves vulnerable to circulating endotoxins and inflammatory mediators [59], and chemotherapy further compromises BNB integrity through mitochondrial dysfunction and endoneurial edema [1]. Thus, the microbiota–nerve axis in CIPN operates through both DRG-mediated (neuronopathy/ganglionopathy) and peripheral nerve-mediated (axonopathy with secondary demyelination) mechanisms, with the relative contribution likely varying by chemotherapeutic class: platinum compounds predominantly cause DRG neuronopathy, while taxanes and vinca alkaloids produce a mixed pattern of DRG injury and distal axonopathy with Schwann cell involvement [23].

### 5.4. Preclinical Evidence of Causality

Microbiota depletion-reduced CIPN. Multiple independent studies demonstrate that antibiotic-mediated gut microbiota depletion prevents or attenuates CIPN. In oxaliplatin-treated mice, antibiotic cocktail feeding prevented thermal hyperalgesia and mechanical allodynia and inhibited proinflammatory cytokine production (TNF-α, IL-1β, IL-6) in DRG. Antibiotic treatment starting simultaneously with chemotherapy produced immediate analgesia [60]. Importantly, antibiotic treatment that was started after establishment of oxaliplatin-induced neuropathic pain did not significantly reverse it, suggesting that gut microbiota may play a more critical role in CIPN initiation than maintenance for this drug class [60].

FMT: transferring pain phenotypes. The most compelling causal evidence comes from reciprocal FMT experiments. Pain-sensitive donor-pain phenotype: Reciprocal microbiota transfers between C57BL/6 (pain-sensitive) and 129SvEv (pain-resistant) mice demonstrated that the gut microbiota determines paclitaxel-induced pain sensitivity. Mice receiving the pain-sensitive B6 microbiota developed CIPN with spinal microglia proliferation, while those receiving the pain-resistant 129 microbiota were protected, with notable absence of infiltrating immune cells [8]. Healthy donor protection: FMT from healthy rats to paclitaxel-treated rats significantly alleviated mechanical allodynia and thermal hyperalgesia, reduced TLR4/p38MAPK pathway activation and astrocytic GFAP expression in the spinal dorsal horn [55]. Restoration of microbiota-pain rescue: In antibiotic-depleted mice protected from CIPN, transplantation of fecal bacteria from specific-pathogen-free mice partially restored the gut microbiota and fully rescued the behaviorally expressed neuropathic pain. *Akkermansia*, *Bacteroides*, and *Desulfovibrionaceae* were identified as taxa potentially playing key roles [60]. In oxaliplatin-treated rats, antibiotic-mediated gut microbiota depletion significantly attenuated neuropathic pain, reduced plasma LPS levels and serum proinflammatory cytokines (TNF-α, IL-6, IL-1β), and suppressed TLR4/MyD88/NF-κB signaling in DRG neurons. Conversely, in the OIPN model, FMT reversed these protective effects, leading to the reappearance of neuropathic pain, increased plasma LPS, decreased intestinal barrier markers, and reactivation of the TLR4/MyD88/NF-κB pathway in DRG [32].

### 5.5. Human Evidence

Microbiota–symptom correlations. Clinical data linking gut microbiota to CIPN in humans remain limited but are growing. In a cohort of 70 early-stage breast cancer patients receiving taxane-based chemotherapy, decreases in microbiota alpha diversity during treatment were associated with worse neuropathy symptoms and general pain after controlling for baseline symptoms. Larger shifts in beta diversity also coincided with more severe neuropathy. SCFA-producing bacteria were depleted in patients with neuropathy at the final infusion, and decreases in fecal SCFA levels correlated with more severe neuropathy symptoms. Circulating neurofilament light chain (NFL), a putative CIPN biomarker, increased during treatment [34]. In breast cancer patients with different paclitaxel-induced neuropathy grades, microbiome and metabolome analysis revealed that severe neuropathy was associated with expansion of *Clostridium* species and elevated DCA levels, linking a specific microbiota–metabolite axis to CIPN severity [48]. In 194 long-term germ cell tumor survivors (median 10-year follow-up), higher plasma HMGB-1 levels (a biomarker of gut microbial translocation) were associated with worse overall CIPN20 scores (*p* = 0.048) and worse motor function (*p* = 0.036), suggesting that gut microbial translocation may contribute to persistent CIPN even years after treatment [47]. A case report of a breast cancer survivor with long-standing CIPN showed that cannabidiol plus multimodal exercise enriched gut genera producing SCFAs and was associated with clinically meaningful improvements in CIPN symptoms and quality of life [61].

## 6. Ozone Therapy: Biological and Molecular Effects

### 6.1. Fundamentals of Ozone Therapy

Ozone as a pro-drug. Ozone (O_3_) is not a drug or medicine in the conventional sense, as it lacks specific cellular receptors and is extremely reactive with biological matrices. Instead, ozone functions as a pro-drug or bioregulator that generates effector molecules upon contact with biological fluids [62]. When gaseous ozone impacts blood or tissues, it is instantly consumed through reactions with polyunsaturated fatty acids (PUFAs) bound to albumin and other biomolecules, producing two classes of messenger molecules that mediate its therapeutic effects [63].

The interaction of ozone with biological substrates generates: (a) Hydrogen peroxide. This represents the early-phase messenger. When ozone reacts with unsaturated fatty acids in aqueous environments (such as lung lining fluids or plasma), the net reaction produces one mole of H_2_O_2_ per mole of ozone consumed, along with two moles of aldehydes. H_2_O_2_ triggers immediate biochemical pathways in blood cells and tissues [63]. (b) Lipid oxidation products (LOPs). These constitute the late-phase messengers and include alkenals (such as 4-hydroxynonenal [4-HNE]), aldehydes, and other lipoperoxides collectively termed “ozonides.” LOPs act as signal transducers that enter cells throughout the body, where they interact with the Nrf2-Keap1 protein complex to initiate adaptive antioxidant responses. The therapeutic dosage range is typically 1–40 μg/mL (depending on protocol), within which ozone triggers a hormetic and adaptive cellular response rather than oxidative damage [64].

### 6.2. Redox Signaling and Hormesis

OT operates through the paradoxical mechanism of hormesis, particularly mitohormesis, whereby low-dose oxidative stress triggers protective cellular adaptations [65].

Activation of Nrf2/HO-1 pathway. The Nrf2 (nuclear factor erythroid 2-related factor 2) pathway is the central mechanism underlying ozone’s therapeutic effects: LOPs, particularly 4-HNE, react with the Nrf2-Keap1 protein complex in the cytoplasm. Under basal conditions, Keap1 sequesters Nrf2 and promotes its degradation. Ozone-derived LOPs modify cysteine residues on Keap1, releasing Nrf2 and allowing its nuclear translocation to chromatin sites of active transcription [66]. Nuclear Nrf2 binds to electrophile-responsive elements (EpRE) in DNA, upregulating expression of antioxidant enzymes (superoxide dismutase [SOD], catalase, glutathione peroxidase (GPx)), phase II detoxification enzymes (NAD(P)H quinone oxidoreductase 1 [NQO1]), and heme oxygenase-1 (HO-1) [67]. In human studies, three sessions of major autohemotherapy (MAH) with ozone (35 μg/mL) significantly increased Nrf2 levels in peripheral blood mononuclear cells immediately after ozone exposure (*p* < 0.01) and 30 min post-reinfusion (*p* < 0.05). After the treatment series, SOD and catalase activities increased (*p* < 0.05), demonstrating sustained antioxidant adaptation [67]. Ozone-induced EpRE activation can be completely reversed by ectopic expression of Keap1, confirming the Nrf2-dependent mechanism [68].

Activation of SIRT1 and related pathways. Ozone activates the AMPK/FOXO/mTOR/SIRT1 pathway, which regulates cellular energy metabolism, autophagy, and longevity [62]. SIRT1 is a NAD^+^-dependent deacetylase involved in mitochondrial biogenesis and stress resistance. The activation of these pathways contributes to ozone’s pleiotropic effects on cellular survival and metabolic adaptation [64]. Antioxidant adaptation. The repetition of graduated oxidative stresses through serial ozone treatments induces a multiform adaptive response (oxidative preconditioning). With progressive OT, the upregulated protective enzymes reverse chronic oxidative stress induced by inflammation, blocking disease progression and improving quality of life. This represents a shift from acute pro-oxidant stimulus to sustained antioxidant capacity—the hallmark of hormesis [65].

### 6.3. Anti-Inflammatory and Immunomodulatory Effects

Reduction in proinflammatory cytokines. OT consistently demonstrates potent anti-inflammatory effects across multiple experimental models. In rheumatoid arthritis models, ozone treatment significantly decreased synovial levels of TNF-α and IL-12p70 while increasing IL-10 (anti-inflammatory cytokine). The most pronounced effects occurred with repeated treatments (at 2, 24, and 48 h post-induction), indicating that sustained OT is more effective than single acute application [69]. In another arthritis model, ozone reduced TNF-α protein concentrations and decreased TNF-α and IL-1β mRNA levels, while reestablishing cellular redox balance and reducing nitric oxide and fructolysine (a marker of glycation) [70]. In psoriasis, topical ozone treatment significantly decreased IL-17A, IL-22, and IL-23 expression in peripheral blood CD4^+^ T cells and inhibited activation of Th17 cells in spleen tissue [71]. In COPD models, ROI decreased secretion of IL-4, IL-17A, TNF-α, IL-1β, and IL-6 in lung tissue [72].

Modulation of the NF-κB pathway. Ozone exerts anti-inflammatory effects through Nrf2/NF-κB crosstalk. Ozone upregulates Nrf2, which in turn inhibits the TLR4/NF-κB pathway. In COPD models, ROI upregulated Nrf2 expression, decreased ROS levels, and downregulated expression of TLR4, NF-κB, and phosphorylated NF-κB (p-NF-κB). When Nrf2 was pharmacologically inhibited (ML385), the anti-inflammatory effects of ozone were abolished, confirming Nrf2-dependent NF-κB suppression [72]. In psoriasis, high-throughput sequencing confirmed that ozone treatment significantly suppressed imiquimod (IMQ)-induced activation of the Toll-like receptor 2 (TLR2)/NF-κB signaling pathway in skin lesions [71]. In neuropathic pain models, intrathecal ozone injection suppressed the GluR6-NF-κB/p65 signaling pathway, reducing spinal expression of IL-1β, IL-6, TNF-α, and NF-κB/p65 [73].

In addition, ozone promotes a cytokine shift from proinflammatory to anti-inflammatory profiles, with initial transient ROS generation followed by sustained anti-inflammatory signaling [74].

### 6.4. Effects on Microcirculation and Oxygen Metabolism

Relevance to peripheral nerves. OT exerts multiple effects on microcirculation and tissue oxygenation that are particularly relevant to peripheral nerve health. Improved microcirculation and perfusion: OT promotes blood circulation and reduces ischemia in both peripheral and central tissues. In peripheral arterial disease (PAD), oxygen-OT improves tissue perfusion, reduces hypoxia, and enhances healing processes. The therapy is effective in low-perfusion syndromes and appears to prevent complications such as amputation in PAD patients [75,76].

Effects on peripheral nerve injury. In a rat sciatic nerve crush injury model, OT (0.7 mg/kg) administered for 4 weeks produced significant histological improvements: reduced nerve diameter and decreased thickness of perineurium and epineurium compared to untreated injury (*p* < 0.001), indicating reduced edema and fibrosis. Decreased vascular congestion and vacuolization (*p* < 0.05), suggesting improved microvascular function. Lower S100 immunoreactivity (a marker of nerve injury and glial activation) compared to untreated injury (*p* < 0.05). Ozone improved sciatic nerve recovery without increasing fibrotic tissue, a critical advantage for functional nerve regeneration [77].

Oxygen metabolism and mitochondrial function. OT targets mitochondria and their turnover/biogenesis, acting as a tuner of fundamental cellular survival mechanisms. Through mitohormesis, ozone enhances mitochondrial structure and function, which is particularly important for energy-demanding peripheral nerves [64]. Ozone indirectly triggers the HIF-1α (hypoxia-inducible factor 1α) pathway, which regulates cellular adaptation to oxygen availability, and activates HO-1 signaling and the NO/iNOS biochemical machinery, all of which contribute to improved tissue oxygenation and vascular function [62].

### 6.5. Clinical Applications in Neuropathic Pain and CIPN

In experimental neuropathic pain models, ozone activates AMPK (AMP-activated protein kinase), which suppresses microglial activation and normalizes pain-related signaling molecules (PKCγ, NMDA receptor, ERK). The anti-nociceptive effect of ozone was abolished by AMPK antagonists, confirming AMPK-dependent mechanisms [78,79].

Overall, OT operates through a sophisticated hormetic mechanism: controlled low-dose oxidative stress generates H_2_O_2_ and LOPs that activate Nrf2/HO-1 and SIRT1 pathways, inducing antioxidant adaptation; suppresses NF-κB-mediated inflammation and proinflammatory cytokines; and improves microcirculation and oxygen metabolism in peripheral nerves. These pleiotropic effects position ozone as a promising adjuvant therapy for CIPN, though robust clinical trial data are still needed [20].

## 7. Clinical Evidence of Ozone Therapy in CIPN

### 7.1. Available Clinical Studies

The clinical evidence for OT in CIPN consists entirely of uncontrolled, preliminary reports from our research group, supplemented by broader cancer survivor studies and a recent narrative review [20].

Case series and retrospective studies directly addressing CIPN. CIPN pain series (2022): a preliminary report of seven patients with chronic pain secondary to grade II–III CIPN treated with ROI. This represents the first published clinical report specifically evaluating OT for CIPN-related pain [80]. CIPN numbness/tingling series (2025): a retrospective study of 15 patients (8 female/7 male, median age 66 years) with persistent numbness and tingling secondary to grade 2–3 CIPN. Ozone was administered by ROI over 40 planned sessions across 4 months, with initial concentration of 10 μg/mL progressively increased to 30 μg/mL and gas volume from 180 mL to 300 mL per session [81].

Broader cancer survivor studies including CIPN patients. Cancer survivor and health-related quality of life (HRQOL) study (2023): a prospective evaluation of 26 cancer survivors with chronic side effects of radiotherapy and chemotherapy (including but not limited to CIPN) assessed health-related quality of life (EQ-5D-5L) and toxicity grade (CTCAE v5.0) before and after OT [82]. Cancer symptom scoping review (2025): a scoping review identified 16 articles evaluating medical ozone treatment for pain, fatigue, anxiety, and depression in cancer patients, providing a broader context for ozone’s role in cancer symptom management [83]. Recent evidence supports OT potential in managing CIPN. In a preliminary clinical report, seven patients with chronic grade II–III CIPN received ozone treatment via ROI. Median pain (Visual Analogue Scale (VAS)) decreased from 7 (baseline) to 4 at treatment end (*p* = 0.004), with sustained improvement at 3 months (VAS 5.5, *p* = 0.008) and 6 months (VAS 6, *p* = 0.008). Half of the patients showed improvement in CTCAE toxicity grade [74]. A 2025 narrative review—the most comprehensive to date—summarized 18 experimental studies and 27 clinical reports (1995–2025) offering preliminary evidence supporting ozone’s role in CIPN management. The hypothesized mechanisms include modulation of oxidative stress, inflammation, microcirculation, and nerve regeneration. However, clinical evidence remains limited, and multiple randomized controlled trials are currently ongoing [20].

### 7.2. Clinical Outcomes

Pain (VAS). In the seven-patient CIPN pain series, ROI produced statistically significant and clinically meaningful pain reduction. Median VAS decreased from 7 (range 5–8) at baseline to 4 (range 2–6) at end of treatment (*p* = 0.004). All patients except one showed clinically relevant pain improvement (defined as ≥2-point VAS reduction) [80]. In the broader cancer survivor cohort (*n* = 26), all dimensions of the EQ-5D-5L questionnaire, including pain/discomfort, were significantly improved (*p* < 0.05), with significant improvement in the overall EQ-5D-5L index (*p* < 0.001) [82]. In a separate case series of six cancer patients with refractory pelvic pain secondary to cancer treatment, VAS decreased from 7.8 ± 2.1 to 2.8 ± 3.8 after 3 months of OT (*p* = 0.020) [20].

Paresthesia (numbness and tingling). The 15-patient retrospective study specifically addressed sensory CIPN symptoms: After ozone treatment, 67% of patients reported a decrease in numbness and tingling of ≥50% (*p* = 0.002) [81]. This is particularly notable given that the ASCO CIPN guideline acknowledges there is no effective clinical management option for numbness and tingling, with duloxetine providing only modest benefit for pain specifically [20,84].

Toxicity grade (CTCAE). OT demonstrated improvement in CIPN toxicity grading: in the pain series, 50% of patients showed improvement in CTCAE v5.0 toxicity grade [80]. In the numbness/tingling series, 47% of patients experienced a decrease in the grade of CIPN toxicity (*p* = 0.016) [81]. In the broader cancer survivor study, the overall grade of toxicity was significantly decreased (*p* < 0.001) [82].

### 7.3. Durability of Response

Six-month follow-up data. Both CIPN-specific studies demonstrated sustained benefit beyond the treatment period in pain outcomes. Median VAS was 5.5 (range 1.8–6.3) at 3 months post-treatment (*p* = 0.008) and 6 (range 2.6–6.6) at 6 months post-treatment (*p* = 0.008), indicating statistically significant improvement persisting at 6 months despite some attenuation from end-of-treatment values [80]. Numbness/tingling outcomes. The improvements in both CIPN toxicity grade and self-reported numbness/tingling were maintained at 3- and 6-month follow-up after the end of ozone treatment [81]. Broader cancer survivor outcomes. In the radiation-induced rectal bleeding series (*n* = 12), OT effects were maintained over a median follow-up of 104 months (range 52–119), suggesting potential for very long-term benefit in some cancer treatment-related toxicities [85].

The durability of response is noteworthy given that ozone treatment was administered over a finite period (~4 months) and benefits persisted after cessation, suggesting that ozone may induce lasting biological changes (e.g., sustained Nrf2-mediated antioxidant adaptation, microbiota remodeling) rather than merely providing symptomatic relief during active treatment [80,81].

### 7.4. Safety Profile

Adverse events. OT by rectal insufflation demonstrates a favorable safety profile across the available literature. In the CIPN studies, no serious adverse events were reported. The most common side effect was soft and temporary flatulence lasting several hours after each session [80,81,85]. In the fibromyalgia rectal insufflation study (24 sessions, 8 mg ozone per session), transient meteorism (abdominal bloating/gas) was the most frequently reported side effect [86]. In the evidence gap map encompassing 26 systematic reviews of OT across multiple indications, no serious adverse effects were reported [87]. When the correct dose is administered within the therapeutic range, no side effects have been reported in neurological applications [75]. In the broader oncology literature, ozone autohemotherapy in 50 cancer patients with fatigue showed no side effects, with 70% achieving significant symptom improvement [88].

Important safety caveats. The therapeutic window is narrow: beneficial hormetic effects (by rectal way or MAH protocol) occur at 10–40 μg/mL, while potential toxicity may occur at doses > 80 μg/mL. This necessitates precise dosing and trained administration [64].

Regulatory considerations. The regulatory status of OT varies significantly across jurisdictions. OT is not officially allowed in many countries, though private medical services use it worldwide. The global legal status of OT is highly polarized. It is a standard complementary practice in parts of Europe and Latin America, but an illegal or unapproved procedure in the US, Canada, and the UK. The primary drivers of legality are not scientific consensus on efficacy, but rather regulatory frameworks regarding natural substances and economic incentives for clinical trials [87].

### 7.5. Critical Appraisal

Absence of microbiome analysis. No clinical study of OT in CIPN has included gut microbiota assessment: despite the strong mechanistic rationale linking ozone and microbiota modulation/SCFA production/neuroprotection, no human study has simultaneously measured microbiota changes and CIPN outcomes during OT [19,20]. The first studies designed to prospectively investigate ROI’s impact on gut microbiota in cancer patients are currently ongoing (NCT07259681 in 38 gynecological cancer patients with pelvic toxicity and NCT06799351 in 42 patients with CIPN). These studies will use 16S rRNA sequencing of stool samples pre- and post-intervention [19]. Without microbiome data, the proposed mechanism linking OT to CIPN improvement via gut microbiota modulation remains entirely hypothetical in the clinical setting, supported only by preclinical evidence and biological plausibility [19].

It is important to note that all published clinical studies on OT in CIPN originate from a single research group. While this reflects the current state of the literature—as no other group has yet published clinical data on this specific indication—it also introduces a potential risk of narrative bias. Independent replication of these findings by other research groups is urgently needed to confirm the observed results and to strengthen the evidence base. The mechanistic framework proposed in this review is therefore presented as a hypothesis-generating synthesis requiring validation through independent studies.

## 8. Ozone Therapy in the Context of Other Microbiome-Modulating Strategies for CIPN: A Hierarchical Appraisal of the Evidence

**Figure 1:**
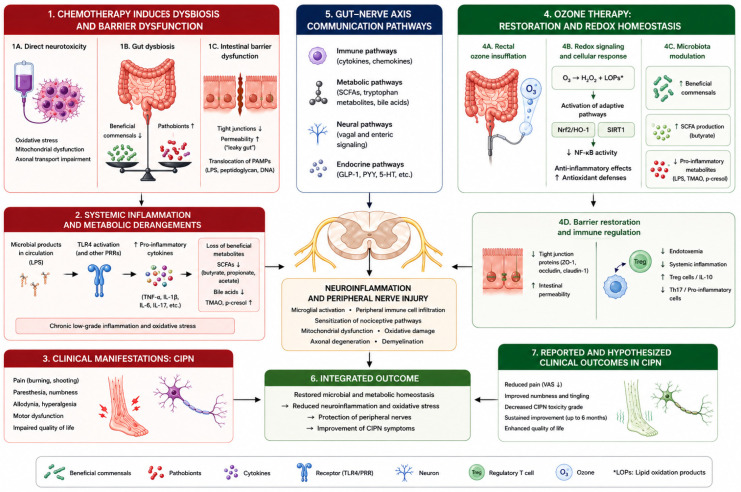
Proposed integrative mechanistic model: ozone therapy, gut microbiota, and CIPN. Abbreviations: CIPN: chemotherapy-induced peripheral neuropathy; SCFAs: short-chain fatty acids; LPS: lipopolysaccharide; TLR4: Toll-like receptor 4; PRRs: pattern recognition receptors; TMAO: trimethylamine N-oxide; O_3_: ozone; H_2_O_2_: hydrogen peroxide; LOPs: lipid oxidation products; Nrf2: nuclear factor erythroid 2–related factor 2; HO-1: heme oxygenase-1; SIRT1: sirtuin 1; NF-κB: nuclear factor kappa B; Treg: regulatory T cells. ↑ Increased; ↓ Decrease; → proposed sequence of pathogenic events; * LOPs and H_2_O_2_ are secondary messengers mediating the biological effects of ozone.

The following integrative model synthesizes the evidence presented in Section 3, Section 4, Section 5, Section 6 and Section 7 into a unified mechanistic framework linking chemotherapy-induced dysbiosis to peripheral neuropathy and proposing OT as a multi-target intervention acting through the gut–nerve axis (Figure 1).

### 8.1. The Pathogenic Cascade: Chemotherapy/Dysbiosis/CIPN

The pathogenic arm of the model follows a sequential, self-amplifying cascade:

Step 1: Chemotherapy-induced dysbiosis. Cytotoxic agents directly damage the intestinal epithelium and profoundly alter gut microbiota composition (see Section 4 for detailed microbial changes), resulting in decreased alpha diversity, loss of SCFA-producing commensals, and expansion of pathobionts, alongside increased intestinal permeability [19].

Step 2: Metabolic and immune derangement. Loss of SCFA-producing bacteria removes anti-inflammatory, epigenetic (HDAC inhibition), and barrier-protective effects, while pathobiont expansion increases production of proinflammatory metabolites including LPS and TMAO that translocate across the compromised intestinal barrier into systemic circulation [49].

Step 3: Neuroinflammation and peripheral nerve damage. Circulating LPS activates the TLR4/NF-κB pathway in DRG neurons and spinal cord microglia, triggering proinflammatory cytokines (TNF-α, IL-1β, IL-6) that sensitize nociceptors, activate spinal glial cells, and damage peripheral nerve fibers (see Section 3 for detailed CIPN pathophysiology). Preclinical evidence demonstrates causality: FMT from pain-sensitive donors transfers the neuropathic phenotype, while FMT from resistant donors confers protection [20,49].

### 8.2. The Therapeutic Intervention: Ozone/Microbiota Restoration/CIPN Improvement

The therapeutic arm proposes OT as a multi-level intervention that reverses the pathogenic cascade at multiple nodes (Figure 1):

**Node 1:** Redox control and hormetic adaptation. ROI generates H_2_O_2_ and LOPs that activate the Nrf2/Keap1/ARE pathway and the SIRT1/AMPK/FOXO axis, shifting the cellular redox environment from oxidative distress toward oxidative eustress (see Section 6.2 for detailed mechanisms) [64,67,68].

**Node 2:** Gut microbiota modulation. The restored redox environment, combined with ozone’s direct antimicrobial effects and enhanced intestinal oxygenation, promotes recolonization by beneficial bacteria (*Lactobacillus*, *Bifidobacterium*, SCFA producers). Preclinical evidence demonstrates that ROI increases fecal butyrate and propionate while decreasing TMAO (see Section 6.4) [3,4,18].

**Node 3:** Intestinal barrier restoration. OT enhances tight junction protein expression and reduces intestinal permeability through Nrf2-mediated cytoprotection and increased butyrate production, preventing LPS translocation and breaking the systemic inflammatory cascade [4,89].

**Node 4:** Anti-inflammatory signaling. Ozone activates Nrf2/NF-κB crosstalk, suppressing TLR4/NF-κB pathway and reducing proinflammatory cytokines (TNF-α, IL-1β, IL-6) while increasing anti-inflammatory mediators (IL-10) (see Section 6.3) [71,72,78,79].

**Node 5:** Gut–nerve axis modulation and neuroprotection. Restored SCFAs—particularly butyrate—exert direct neuroprotective effects via GPR41/GPR43 activation, HDAC inhibition, and enhanced peripheral nerve microcirculation, providing a biological rationale for clinical improvement in pain, paresthesia, and CIPN toxicity grade [77,80,81].

### 8.3. Evidence Strength, Knowledge Gaps, and Distinction Between Established Evidence and Hypotheses

The strength of evidence varies substantially across the proposed mechanistic nodes, and it is critical to distinguish clearly between (i) established experimental findings, (ii) mechanistically coherent but untested hypotheses, and (iii) areas that remain entirely unexplored.

**i. Established evidence.** The following components of the model are supported by direct experimental data:

Ozone activates Nrf2/HO-1 and suppresses NF-κB, as demonstrated in preclinical models and in human peripheral blood mononuclear cells (PBMCs) [67,68,72].

ROI increases Lactobacillus, Bifidobacterium, and fecal SCFAs while decreasing trimethylamine N-oxide (TMAO), as shown in ApoE^−/−^ mice [67,68,72].

Ozone restores intestinal barrier function in a microbiota-dependent manner, confirmed by antibiotic depletion experiments [18].

Butyrate from butyrate-producing bacteria such as Roseburia intestinalis directly alleviates neuropathic pain via GPR41/GPR43 signaling in preclinical models [3,18,49].

**ii. Mechanistic hypotheses requiring direct validation.** The following proposed links, while biologically plausible and mechanistically coherent, have never been tested as integrated sequences:

That ozone-induced changes in microbiota composition translate into measurable neurological improvement in CIPN.

That the anti-inflammatory and neuroprotective effects of ozone in CIPN are mediated specifically through microbiota-dependent pathways (rather than through direct systemic effects).

That the self-amplifying pathogenic loop—dysbiosis, barrier dysfunction, inflammation, oxidative stress, worsened dysbiosis—is effectively interrupted by ozone at multiple nodes simultaneously in the context of CIPN.

**iii. Critical knowledge gaps.** Several gaps must be acknowledged before this model can be considered clinically relevant:

The complete integrated pathway—from OT through microbiota modulation to CIPN improvement—has never been tested as a unified hypothesis, either preclinically or clinically [81].

Clinical CIPN studies to date have not included longitudinal microbiome profiling, and preclinical microbiome studies have not used CIPN models [81].

The optimal ozone administration protocol (dose, frequency, duration, route) for microbiota modulation in the context of CIPN remains undefined.

No study has directly demonstrated that ozone-induced microbiota changes correlate with clinical outcomes in CIPN patients.

**Ongoing and future research.** Two ongoing clinical studies represent the first attempt to bridge these gaps by simultaneously measuring microbiota changes and clinical outcomes during OT in cancer patients with CIPN (NCT06706544) and in patients with radiotherapy/chemotherapy-induced pelvic toxicity (NCT07259681) [19]. Until their results are available, and until mechanistic preclinical studies confirm the microbiota-dependence of ozone’s neuroprotective effects, the integrative model presented here should be regarded as a hypothesis-generating framework rather than a basis for clinical practice.

### 8.4. Evidence Hierarchy and Limitations

This evidence hierarchy reveals that the proposed microbiota–OT–CIPN pathway rests on a foundation of strong preclinical evidence (Level I) and biologically plausible hypotheses (Level II), but is supported by weak clinical evidence (Level III) with critical gaps (Level IV) (Table 3). Until Levels III and IV are addressed through rigorously designed RCTs with integrated microbiome endpoints, the integrative model presented here should be regarded as a hypothesis-generating framework rather than a basis for clinical practice. This hierarchical appraisal is essential for readers to appropriately calibrate their interpretation of the proposed therapeutic rationale.

### 8.5. Ozone Therapy in the Context of Other Microbiome-Modulating Strategies for CIPN

To provide a balanced perspective, it is important to compare OT with other microbiota-targeted interventions that have been investigated in the context of CIPN and chemotherapy-induced dysbiosis. While direct head-to-head comparisons are lacking, the following analysis highlights the relative evidence base, advantages, and limitations of each approach.

FMT has demonstrated efficacy in multiple preclinical CIPN models. In paclitaxel-induced peripheral neuropathy (PIPN) rats, FMT from healthy donors significantly alleviated mechanical allodynia and thermal hyperalgesia, suppressed TLR4/p38MAPK pathway activation, and reduced astrocyte activity [55]. In oxaliplatin-induced peripheral neuropathy (OIPN), antibiotic-mediated microbiota depletion attenuated neuropathic pain and reduced plasma LPS, while FMT reversed these protective effects, reactivating the TLR4/MyD88/NF-κB pathway in DRG neurons [32]. Bidirectional FMT experiments have further shown that transplantation of dysbiotic microbiota into healthy animals induces pain-like hypersensitivity and neuroinflammation via IBA1, TNF-α, and IL-1β upregulation, while healthy donor FMT restores claudin-5, anti-inflammatory markers (TGF-β, IL-10), and downregulates pain-related ion channels (TRPM8, Nav1.8, Nav1.7, TRPA1) [90]. In chemotherapy-induced dysbiosis models, FMT reversed antibiotic- and 5-FU-induced dysbiosis, restoring anti-inflammatory species including *Clostridium scindens* and *Faecalibacterium prausnitzii* [91].

Probiotics and microbial metabolites: Emerging evidence supports specific microbial interventions for CIPN. Combined administration of *Akkermansia muciniphila* and sodium butyrate exerted potent neuroprotective effects in OIPN rats, attenuating mechanical and cold allodynia, ameliorating DRG neuronal atrophy and axonal degeneration, preserving IENFD, modulating inflammatory cytokines (↓IL-6, IL-1β, TNF-α; ↑IL-10), and reducing serum NFL—establishing NFL as an objective biomarker for monitoring OIPN severity and therapeutic response. Combination therapy was superior to either agent alone [92]. Sodium butyrate alone demonstrated neuroprotection via anti-inflammatory (↓TNF-α, ↑IL-10), neurotrophic (↑NGF), and immunomodulatory (↑FOXP-3, ↑PPAR-γ) mechanisms, with prophylactic administration showing greater efficacy than concurrent treatment [51].

Prebiotics and dietary interventions: Dietary modulation represents a low-risk, accessible strategy with emerging evidence. Observational data in cancer survivors suggest that higher diet quality (Healthy Eating Index) is associated with lower CIPN severity (OR = 0.94, *p* = 0.03), with refined grain intake increasing risk (OR = 1.11, *p* = 0.04) and fish (OR = 0.21), eggs (OR = 0.06), and selenium (OR = 0.96) being protective [93,94]. Dietary bioactive compounds including curcumin, resveratrol, omega-3 PUFAs, epigallocatechin gallate, and naringin have demonstrated anti-neuropathic effects in preclinical models via NF-κB/MAPK suppression, cannabinoid receptor activation, and sodium channel modulation [95]. However, no clinical trial has specifically evaluated a prebiotic or dietary fiber intervention for CIPN prevention or treatment, and this approach requires dedicated investigation.

Comparative evidence gaps. Direct comparisons between these strategies are unavailable. No study has compared ROI to FMT, probiotics, or prebiotics in CIPN. Furthermore, the evidence base differs substantially: FMT and probiotics have been tested specifically in CIPN models, whereas the preclinical evidence for ROI in CIPN is indirect, derived primarily from non-CIPN disease models. The ongoing RCT OzoParQT (NCT06706544) and its ancillary microbiome study (NCT06799351) represent the first attempt to generate clinical evidence on ROI with integrated microbiome profiling [93].

Combination and complementary approaches: An important consideration is that these strategies are not mutually exclusive. ROI’s proposed dual mechanism—combining microbiota modulation with systemic Nrf2-mediated hormesis—could theoretically complement direct microbiota-targeted interventions such as probiotics or dietary modifications. For example, ROI-induced enhancement of intestinal barrier function and redox balance could create a more favorable ecological niche for subsequently administered probiotics or dietary SCFA precursors. Conversely, prebiotic or dietary interventions that increase SCFA-producing bacteria could potentiate the microbiota-dependent component of ROI’s effects. However, no study has evaluated any combination of these strategies, and such approaches remain entirely speculative.

## 9. Clinical Translation and Future Directions

Three priorities will determine whether the hypothesis presented in this review advances from theoretical framework to clinical reality:

1. Completion of the first RCT of OT for CIPN. A sham-controlled, double-blind randomized controlled trial with integrated microbiome and metabolomic endpoints, using validated CIPN assessment instruments (e.g., EORTC QLQ-CIPN20, VAS for pain, and CTCAE v5.0 for toxicity grading), is urgently needed to establish efficacy and causality. The ongoing OzoParQT trial (NCT06706544) represents the first attempt to address this gap.
2. Validation of a multi-modal biomarker panel. A composite biomarker panel—including microbiota composition (16S rRNA sequencing), fecal SCFAs, serum NFL and inflammatory markers (IL-6, TNF-α, LPS)—should be validated for its capacity to track the gut–nerve axis in real time during chemotherapy and intervention. Such a panel would enable early detection of dysbiosis, prediction of CIPN risk, and monitoring of therapeutic response.
3. Mechanistic confirmation through preclinical studies. Direct preclinical testing is required to determine whether ozone’s neuroprotective effects in CIPN models are: (i) abolished by antibiotic-mediated microbiota depletion; and (ii) recapitulated by SCFA supplementation alone. Such studies would establish whether the microbiota is a necessary mediator of ozone’s effects or merely a correlate.

Until these milestones are achieved, the integrative model presented here should be regarded as a research framework and hypothesis-generating synthesis rather than a basis for clinical practice. The convergence of microbiome science, redox biology, and neuroimmunology has created an unprecedented opportunity to address one of supportive oncology’s most persistent unmet needs—but realizing this opportunity demands the rigorous, well-designed clinical investigation that the hypothesis deserves and the patients require [96].

## 10. Conclusions

This article has examined the convergence of three rapidly evolving fields—CIPN, gut microbiota science, and OT—to construct an integrative mechanistic hypothesis proposing that OT may ameliorate CIPN through modulation of the gut–nerve axis. It is important to emphasize that this hypothesis is built upon preclinical evidence and small, uncontrolled clinical case series (*n* = 7 and *n* = 15); the clinical data currently available are insufficient to establish efficacy or to support any therapeutic recommendation.

The proposed model rests on a logical chain of evidence assembled from independent research streams that, while individually substantiated, have never been tested as a unified pathway in a single study. Mechanistic coherence does not constitute proof of efficacy, and the complete chain—from ozone administration through microbiota modulation to measurable CIPN improvement—remains untested as an integrated sequence.

We wish to emphasize explicitly that the biological plausibility outlined in this article should not be interpreted as clinical evidence supporting the use of ozone therapy in patients with CIPN. No clinical recommendation can be made on the basis of the current evidence, and any therapeutic application outside of approved clinical trials would be premature. Until rigorous RCTs with integrated microbiome endpoints are completed, the framework presented here should be regarded as hypothesis-generating rather than a basis for clinical practice.

## Figures and Tables

**Table 1:** Drug–mechanism relationship in axonal injury [1,7,21,22].

Drug Class	Key Mechanism	Axonal Effect
Taxanes	Microtubule stabilization	Transport blockade, dying-back
Platinum compounds	DNA adducts (including mitochondrial)	DRG apoptosis, fiber degeneration
Vinca alkaloids	Tubulin polymerization blockade	Microtubule and transport disruption

Legend: DRG: dorsal root ganglion.

**Table 2:** The effects of selected chemotherapeutic agents on gut microbiota alterations [32,33,34].

Drug	Bacteria Decreased	Bacteria Increased/Pathobionts Expanded	Specific Dysbiosis Mechanism	Associated GI Toxicity
Oxaliplatin (L-OHP)	*Bifidobacterium*, *Akkermansia*, *Allobaculum*, *Lactobacillus*	*Proteobacteria*, *Escherichia-Shigella*, *Proteus*, *Helicobacter*, *Streptococcus*	Causes the most severe intestinal damage and greatest microbial disorder; considerable overlap with 5-FU and irinotecan dysbiosis patterns; maximal NOD/RIP2/NF-κB activation; kynurenine (via IDO1/IFNγ) mediates *L. johnsonii* loss and activates TNF-α/JNK	Severe diarrhea, mucositis, ROS- and cytokine-mediated hyperalgesia
Cisplatin	*Ruminococcus gnavus*, *Firmicutes* (selectively), *Lactobacillus*	*Proteobacteria*, *Bacteroidetes*	Direct antibiotic-like activity; selective depletion of protective strains (*R. gnavus*); extensive mucosal damage with bacterial translocation; dysbiosis is more persistent than with other agents	Mucositis, chronic mucosal damage, reduced mucin production, systemic inflammation (↑IL-6)
Paclitaxel/Nab-paclitaxel	*Actinobacteriota*, *Firmicutes* (progressively with consecutive cycles)	*Proteobacteria*, *Cyanobacteria* (increase with successive cycles)	Prolonged proinflammatory circulating and central signals; dysbiosis accumulates with consecutive cycles; alters microbiota–gut–liver axis affecting pharmacokinetics (↑AUC, ↑Cmax) through non-CYP-dependent mechanisms	Diarrhea, nausea/vomiting; neurological effects mediated by microbiota–gut–brain axis
Vincristine/Vinca alkaloids	*Bacteroides*, *Clostridium cluster XIVa*, *Faecalibacterium prausnitzii*, *Bifidobacterium*, *Lachnospiraceae* (in ALL induction regimens containing VCR)	*Enterococcus* spp. (dramatic increase; dominance ≥ 30% predicts febrile neutropenia), *Ruminococcus gnavus*, *R. torques*, *Enterobacteriaceae*	Primary mechanism is neurotoxic, not directly cytotoxic to epithelium: disrupts microtubules in enteric neurons → myenteric plexus injury via pro-inflammatory macrophage activation (ERK1/2, p38-MAPK) → ↑IL-1β, IL-6, TNF-α → severe dysmotility/ileus → secondary stasis-driven dysbiosis; also disrupts GI regulatory peptide cells (gastrin, somatostatin, 5-HT, enteroglucagon); ↑ROS and ↓Nrf2 in colonic tissue	Constipation (most characteristic; upper-colon impaction), paralytic ileus, intestinal necrosis/perforation (rare), abdominal pain; CIPN via microbiota–gut–brain axis

Abbreviations: AUC: area under the curve; Cmax: maximum plasma concentration; GI: gastrointestinal; IDO1: indoleamine 2,3-dioxygenase 1; IFNγ: interferon gamma; IL: interleukin; JNK: c-Jun N-terminal kinase; MAPK: mitogen-activated protein kinase; NF-κB: nuclear factor kappa-light-chain-enhancer of activated B cells; NOD: nucleotide-binding oligomerization domain; RIP2: receptor-interacting serine/threonine-protein kinase 2; ROS: reactive oxygen species; TNF-α: tumor necrosis factor alpha; VCR: vincristine. ↑ Increased; ↓ Decrease; → proposed sequence of pathogenic events.

**Table 3:** Hierarchical summary.

Level	Evidence Type	Strength	Key Limitation
I	Established experimental findings	Strong	Preclinical only; translatability to humans unknown
II	Mechanistic hypotheses	Plausible	Never tested as integrated sequences
III	Clinical evidence	Weak	Small, uncontrolled, single-group case series
IV	Knowledge gaps	Critical	No unified pathway tested; no microbiome data in clinical studies
V	Ongoing research	Pending	Results not yet available

## Data Availability

No new data were created or analyzed in this study.

## Abbreviations

The following abbreviations are used in this manuscript:

4-HNE	4-hydroxynonenal
AMPK	AMP-activated protein kinase
BNB	Blood–nerve barrier
CIPN	Chemotherapy-induced peripheral neuropathy
CTCAE	Common Terminology Criteria for Adverse Events
DCA	Deoxycholic acid
DRG	Dorsal root ganglion
FMT	Fecal microbiota transplantation
GALT	Gut-associated lymphoid tissue
GenAI	Generative artificial intelligence
HDAC	Histone deacetylase
HIF-1α	Hypoxia-inducible factor 1-alpha
HO-1	Heme oxygenase-1
H_2_O_2_	Hydrogen peroxide
IL	Interleukin
IENFs	Intraepidermal nerve fibers
LPS	Lipopolysaccharide
LOPs	Lipid oxidation products
MAH	Major autohemotherapy
MAPK	Mitogen-activated protein kinase
MOR	μ-opioid receptor
NaB	Sodium butyrate
NFL	Neurofilament light chain
NF-κB	Nuclear factor kappa-light-chain-enhancer of activated B cells
Nrf2	Nuclear factor erythroid 2-related factor 2
NTS	Nucleus tractus solitarius
OIPN	Oxaliplatin-induced peripheral neuropathy
OT	Ozone therapy
PAD	Peripheral arterial disease
PAMP	Pathogen-associated molecular pattern
PBMC	Peripheral blood mononuclear cell
PIPN	Paclitaxel-induced peripheral neuropathy
PNS	Peripheral nerve system
PUFA	Polyunsaturated fatty acid
RCT	Randomized controlled trial
ROI	Rectal ozone insufflation
ROS	Reactive oxygen species
SCFAs	Short-chain fatty acids
SIRT1	Sirtuin 1
SOD	Superoxide dismutase
TLR4	Toll-like receptor 4
TMAO	Trimethylamine N-oxide
TNF-α	Tumor necrosis factor alpha
VAS	Visual Analogue Scale
ZO-1	Zonula occludens
